# Enhanced Pelican Optimization Algorithm with Deep Learning-Driven Mitotic Nuclei Classification on Breast Histopathology Images

**DOI:** 10.3390/biomimetics8070538

**Published:** 2023-11-10

**Authors:** Fadwa Alrowais, Faiz Abdullah Alotaibi, Abdulkhaleq Q. A. Hassan, Radwa Marzouk, Mrim M. Alnfiai, Ahmed Sayed

**Affiliations:** 1 Department of Computer Sciences, College of Computer and Information Sciences, Princess Nourah Bint Abdulrahman University, P.O. Box 84428, Riyadh 11671, Saudi Arabia; 2 Department of Information Science, College of Humanities and Social Sciences, King Saud University, P.O. Box 28095, Riyadh 11437, Saudi Arabia; 3 Department of English, College of Science and Arts at Mahayil, King Khalid University, Abha 62529, Saudi Arabia; 4 Department of Information Systems, College of Computer and Information Sciences, Princess Nourah bint Abdulrahman University, P.O. Box 84428, Riyadh 11671, Saudi Arabia; 5 Department of Information Technology, College of Computers and Information Technology, Taif University, Taif P.O. Box 11099, Taif 21944, Saudi Arabia; 6 Research Center, Future University in Egypt, New Cairo 11835, Egypt

**Keywords:** medical imaging, artificial intelligence, bio-inspired algorithm, mitotic nuclei classification, deep learning

## Abstract

Breast cancer (BC) is a prevalent disease worldwide, and accurate diagnoses are vital for successful treatment. Histopathological (HI) inspection, particularly the detection of mitotic nuclei, has played a pivotal function in the prognosis and diagnosis of BC. It includes the detection and classification of mitotic nuclei within breast tissue samples. Conventionally, the detection of mitotic nuclei has been a subjective task and is time-consuming for pathologists to perform manually. Automatic classification using computer algorithms, especially deep learning (DL) algorithms, has been developed as a beneficial alternative. DL and CNNs particularly have shown outstanding performance in different image classification tasks, including mitotic nuclei classification. CNNs can learn intricate hierarchical features from HI images, making them suitable for detecting subtle patterns related to the mitotic nuclei. In this article, we present an Enhanced Pelican Optimization Algorithm with a Deep Learning-Driven Mitotic Nuclei Classification (EPOADL-MNC) technique on Breast HI. This developed EPOADL-MNC system examines the histopathology images for the classification of mitotic and non-mitotic cells. In this presented EPOADL-MNC technique, the ShuffleNet model can be employed for the feature extraction method. In the hyperparameter tuning procedure, the EPOADL-MNC algorithm makes use of the EPOA system to alter the hyperparameters of the ShuffleNet model. Finally, we used an adaptive neuro-fuzzy inference system (ANFIS) for the classification and detection of mitotic cell nuclei on histopathology images. A series of simulations took place to validate the improved detection performance of the EPOADL-MNC technique. The comprehensive outcomes highlighted the better outcomes of the EPOADL-MNC algorithm compared to existing DL techniques with a maximum accuracy of 97.83%.

## 1. Introduction

On a global scale, women are majorly impacted by breast cancer (BC), and it is the second major cause of death after lung tumours. Ductal carcinoma in situ (DCIS) is a common type of BC that occurs in milk ducts and cannot disseminate to other tissues [1]; invasive ductal carcinoma (IDC) can also occur in milk ducts, however may also spread to other nearby tissues; tubular carcinoma is a subcategory of IDC; medullary carcinoma can be a heavy mass; and invasive lobular carcinoma (ILC) begins at the lobules for milk production [2]. Various methods, namely biopsy, mammography, magnetic resonance imaging (MRI), and ultrasound, can be employed to analyse these diseases [3]. In a condition of biopsy, a part of the tissue is removed from the cancerous region, employing surgical procedures or other techniques, namely fine needle aspiration (FNA), which is further utilized to make a slide. Haematoxylin and Eosin (H&E) stains have been employed on the tissues, but Haematoxylin turns nuclei blue, whereas the cytoplasm acquires a pink colour because of Eosin [4]. WSI scanner has been utilized for integrating every analysis of a tissue on a microscope (called High Power Fields (HPF)) into a single image named Whole Slide Image (WSI). These images are exploited for further examination through image processing methods and are simply transmitted and stored through the internet [5].

The number of mitoses is recognized as a better predictor by pathologists to measure the aggressiveness of cancer [6]. Several cells divide meaning the cancer can be highly aggressive. Mitosis includes four primary stages and the shape of a nucleus at every stage moderately varies; however, it is more closely similar to the non-MCs. The count of mitotic cells (MCs) in HI is one of the major three elements (the other two being nuclear pleomorphism and tubule development) needed to develop computer-aided categorization of BC tissue images [7]. This can be extremely difficult because the biological variability of the MCs causes their highly complex identification. In addition to the development of digital pathology, numerous computational systems are designed for automating pathological efforts [8]. Current developments in deep convolutional neural networks (DCNNs) and their desirable execution on image classification, identification, and segmentation are augmented by their utilization in medical imaging issues. Recently, artificial intelligence (AI) methods have had a significant effect on all domains of life and even in the medical domain [9]. Most of the techniques have been recently automated and even utilized as a second-opinion method in medical analysis. AI algorithms were designed earlier to solve difficulties in the healthcare sector [10]. The identification of MC is automated by employing AI methods, but it contains numerous problems. For instance, it is hard to distinguish between normal cells’ and MCs’ absence of pathological information and the applications of higher-resolution microscopes due to MCs’ morphological and texture features, which can be the same as in healthy cells.

In this article, we present an Enhanced Pelican Optimization Algorithm with Deep Learning-Driven Mitotic Nuclei Classification (EPOADL-MNC) technique on Breast Histopathology Images. This developed EPOADL-MNC method examines the histopathology images for the classification of MCs and non-MCs. In the presented EPOADL-MNC technique, the ShuffleNet model can be employed for the feature extraction method. For the hyperparameter tuning procedure, the EPOADL-MNC model makes use of the EPOA system to adjust the hyperparameters of the ShuffleNet model. Finally, we used an adaptive neuro-fuzzy inference system (ANFIS) for the classification and detection of mitotic cell nuclei on histopathology images. A series of simulations took place to validate the improved detection performance of the EPOADL-MNC technique. In short, the key contribution of the paper is summarized as follows:

- An automated EPOADL-MNC technique comprising a ShuffleNet feature extractor, EPOA-based hyperparameter tuning, and ANFIS classification model for mitotic nuclei classification has been developed. To the best of our knowledge, the EPOADL-MNC technique has never existed in the literature.
- This paper leverages the ShuffleNet model for feature extraction, which enables the algorithm to learn intricate hierarchical features from histopathology images, improving its ability to detect subtle patterns related to mitotic nuclei.
- The EPOADL-MNC employs the EPOA for fine-tuning the hyperparameters of the ShuffleNet model. This optimization procedure enhances the model’s performance and adaptability. Hyperparameter optimization using the EPOA algorithm and using cross-validation helps to boost the predictive outcome of the EPOADL-MNC model for unseen data.
- This paper utilizes the ANFIS model for the final classification and detection of mitotic cell nuclei in histopathology images. ANFIS combines fuzzy logic and neural networks for accurate classification.

## 2. Related Works

Bhausaheb and Kashyap [11] designed a BC classification technique called the Shuffled Shepherd Deer Hunting Optimizer-assisted DNN (SSDHO-based DNN) algorithm. This method, SSDHO, has been designed by combining the Shuffled Deer Hunting Optimization Algorithm (DHOA) and Shepherd Optimizer Algorithm (SSOA). Now, CNN, shape, and statistical features have been efficiently extracted from the segmentation of blood cells. Sohail et al. [12] designed a novel DCNN-based Heterogeneous Ensemble approach “DHE-Mit-Classifier”. Primarily, mitotic patches were classified and identified into non-mitotic and mitotic nuclei employing this developed DHE-Mit-Classifier. The five different DCNNs have been developed and employed as base classifiers. Multi-layer perceptron (MCP) was utilized as a meta-classifier to establish an accurate and robust classifier. In [13], an optimization-based superpixel-clustering framework was presented. With the help of superpixel with Grey Wolf Optimizer (GWO) and PSO methods, segmentation could be executed following the normalization. Subsequently, feature extraction has been performed by exploiting perimeter, eccentricity, circularity, colour autocorrelogram, GLCM, Local Direction Ternary Pattern (LDTP), and solidity. Eventually, the SVM technique can be employed.

Sampath and Srinath [14] introduced a novel technique named “Hybrid CNN”, integrating the Sine Cosine Algorithm (SCA) in addition to the TL method. This framework utilizes a TL approach. The hyperparameters are established by applying SCA, which can be employed in the VGG16 framework. ImageNet was implemented for pre-training the network and the final three convolutional layers that can be trained using the TL method. Wang et al. [15] implemented a BC-HPI classification that depends on deep-FF and an improved routing (FE-BkCapsNet) framework. Initially, a new architecture with dual channels incorporating spatial and semantic features into novel capsules was developed. Subsequently, routing coefficients have been enhanced adaptably and indirectly by adjusting the loss function as well as embedding the routing method into a complete optimizer approach. In [16], hand-crafted feature extraction methods (colour histogram, Haralick textures, and Hu moment) and the DNN algorithm are utilized for multi-classification BC, applying HPIs on the BreakHis database. The feature extraction utilizing the hand-crafted methods was implemented for training the DNN algorithms with SoftMax and four dense layers. Kausar et al. [17] implemented a novel DL-based algorithm for breast H&E-stained breast HPIs. In this work, the colour normalization technique and feature extraction scheme were utilized for pre-processing. Additionally, deep feature computation with DCNN and classification with different deep classifiers are also described. Botlagunta et al. [18] presented a non-invasive BC classification approach. To determine the statistical importance of the databases, the Welch Unpaired t-test is exploited. Text-mining architecture from EMR makes it simpler to discrete the blood profile information and recognize MBC patients.

Huang et al. [19] present a new multi-task segmentation network which combines background and contour segmentation with the nuclei segmentation process and generates further accurate segmentation outcomes. The convolutional and attention components have been combined with the model to enhance its global focus and improve segmentation outcomes indirectly. Wang et al. [20] examine a generalizable and robust mitosis detection approach (named FMDet) that is independently tested on multi-centre breast histopathological images. To capture additional refined morphological features of cells, the authors transform the object detection task as a semantic segmentation issue. In [21], the SMDetector is presented, and it is a DL approach used to detect smaller objects like mitotic and non-mitotic nuclei. This method utilizes dilated layers from the backbone to prevent smaller objects from disappearing in the deep layers.

A significant research gap in the domain of DL-driven mitotic nuclei classification on breast histopathology images is the imperative need for systematic hyperparameter tuning. DL models are highly sensitive to the values of hyperparameters, including learning rates, batch sizes, and network architectures. In the context of mitotic nuclei classification, the optimal combination of hyperparameters can dramatically impact the model’s ability to detect and classify these crucial cellular structures accurately. Effective hyperparameter tuning methods tailored to the nuances of the histopathology image analysis task are essential to unlock the full potential of DL algorithms, and addressing this gap is paramount for achieving the highest levels of accuracy and reliability in breast cancer diagnosis.

## 3. The Proposed Model

**Figure 1:**
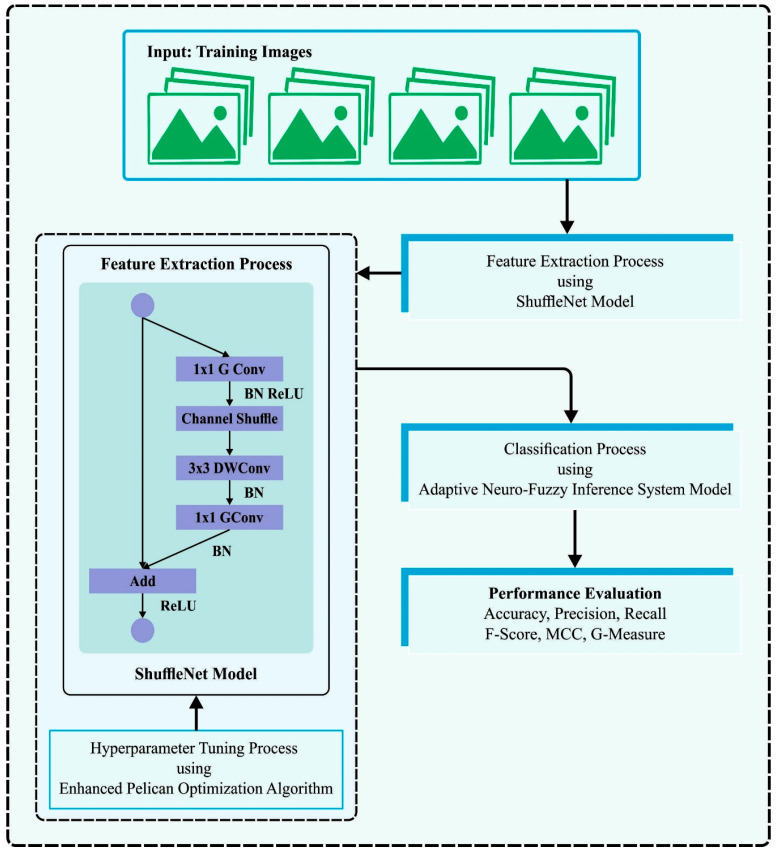
Workflow of EPOADL-MNC system.

In this study, we have presented the EPOADL-MNC system for automated mitotic nuclei classification. A goal of the presented EPOADL-MNC algorithm depends on the examination of the histopathology images for the classification of MC and non-MCs. In the presented EPOADL-MNC technique, the ShuffleNet feature extractor, EPOA-based parameter tuning, and ANFIS classifier are involved. Figure 1 depicts the workflow of the EPOADL-MNC algorithm.

### 3.1. Stage I: ShuffleNet Model

The ShuffleNet architecture can be employed for processing feature extraction. We applied the ShuffleNet, and a remarkably efficient DL structure was implemented. Based on the hardware (computational resource), we employed the ShuffleNetv1 version of the pretrained ShuffleNet architecture to obtain improved results with lower computation costs [22]. In contrast with typical CNN, the presented architecture is deeper than with fifty learnable layers, viz., an FC layer, one convolution (Conv) layer, and forty-eight group convolutional layers. The framework has an overall 172 layers, containing 49 BN layers, one maximum pooling layer, four average pooling layers, 33 rely layers, a classification layer, and a SoftMax layer. The architecture uses four pooling layers to lessen the computation difficulty.

The initial layer is the input layer, and input images of size 224 × 224 (ECG trace image, chest radiograph, and CT scan) are used for processing. To create the mapping feature, the initial Conv layer is applied to extract the features at the input image of size 224 × 224 by using 24 filters (kernels) of size 3 × 3 with a stride of 2×2. The output of the mapping feature (Conv layers) is computed by using Equation (1):

(1) $$s\left(i,j\right)=\left(I\times K\right)\left(i,j\right)=\sum_{n}^{}\sum_{m}^{}I\left(m,n\right)K\left(i-m,j-n\right)$$

where the kernel of the existing Conv layer is K. The output feature mapping can be represented as s, and the input image can be represented as i. The output of size o=((i−k)+2p)/(s+1) is generated after using an operation on the input images, where i refers to the input, p means padding, and k denotes kernel size.

The ShuffleNet component with a stride (shift) of 2 × 2 provides resultant feature maps of the initial Conv layer. The ShuffleNet module includes three Conv functions, namely 3 × 3 depth-wise convolutions and two 1 × 1 point-wise group convolutions. Followed by channel shuffle operation, BN, and ReLU function, the initial point-wise group convolution is performed. ReLU function is used since it is straightforward and efficient.

(2) $$f\left(x\right)=\left\{\begin{array}{l} 0,x<0\\ x,x\leq 0 \end{array}\right.$$

ReLU is used to deactivate the neuron (set neurons to 0) at negative values and activate neurons with positive values. Followed by BN, the second and third Conv functions include 3 × 3 depth-wise convolutional layers and a 1 × 1 point-wise group convolutional layer. On the shortcut path, the architecture has a three-by-three average pooling. The module comprises 16 successive ShuffleNet modules and 50 layers, all of which provide trained feature maps. In addition, this layer performs feature extraction. This feature map is submitted to FC, and SoftMax activation is used for determining the classification probability utilized by the last classification layer. The working of the FC layer is shown in Equation (3).

(3) $$a_{i}=\sum_{j=0}^{m\times n-1}w_{ij}\times x_{i}+b_{i}$$

In Equation (3), the depth height, index of FC, and width layers are represented as d, n, i, and m. The output index of the FC layer is i. Moreover, b and w characterize the bias and weights, correspondingly.

### 3.2. Stage II: EPOA-Based Hyperparameter Optimizer

The EPOA-based hyperparameter tuning process is implemented for the ShuffleNet model. POA emulates the evolutionary process in an ecosystem by considering pelicans as individual members of the population [23]. Every member provides optimization suggestions and represents a potential solution, which is based upon setting the difficult variables to the position of every pelican from the search region. During the initialization of the population, every individual is initialized arbitrarily within the lower as well as upper boundaries of the search problem to ensure the global searching capability and the diversity of the population as follows.

(4) $$x_{\left(i,j\right)}=l_{j}+rand\cdot \left(u_{j}-l_{j}\right),i=1,2,\ldots ,N,j=1,2,\ldots ,m$$

In Equation (4), the variable $\chi_{i,j}$ denotes the value of the $j^{th}$ parameter in the $i^{th}$ candidate solution. N refers to the overall amount of individuals in the population. m is the no. of problem variables, representing a number of parameters or features to be enhanced. $l_{j}$ and $u_{j}$ variables are the lower as well as upper boundaries of the $j^{th}$ variables, correspondingly, and such boundaries are substantial to control the range of solution space. 

The POA stimulates the behaviours and tactics that pelicans use while hunting and attacking the target to update possible solutions. Initially, the tactics through which pelican members approach once they spot prey are simulated as follows.

(5) $$x_{i,j}^{P_{1}}=\left\{\begin{array}{l} x_{i,j+rand\cdot (p_{j}-I\cdot x_{i,j})},\;if\;F_{p}<F_{i}\\ x_{i,j+rand\cdot \left(x_{i,j}-p_{j}\right)},\;else \end{array}\right.$$

Based on Equation (6), we can observe the significance of parameter $x_{i}^{P_{1}}$, representing the updated position of the pelican in the *j^th^* dimension owing to this stage 1 that can be $i^{th}$ pelicans. I is a random integer that ranges within [0, 1]. Moreover, the parameter $p_{j}$ is used to represent the location of prey in the $j^{th}$ parameter, and $F_{p}$ shows the value of the objective function. By integrating Equation (6), we are capable of simulating and modelling these processes effectively.

(6) $$X_{i}=\left\{\begin{array}{ll} X_{i}^{P_{1}}, & if\;F_{i}^{P_{1}}<F_{i}\\ X_{i}, & else \end{array}\right.$$

where the objective function value obtained during stage 1 is represented as $F_{i}^{P_{1}}$, and the updated status of the $i^{th}$ pelican after stage 1 is $X_{i}^{P_{1}}$.

The computed formula gives the means to analyse and simulate the hunting behaviours of individual pelicans. When collected through the pouch on its neck, the individual pelican works as a storing function for fish when hunting begins.

(7) $$x_{i,j}^{P_{2}}=x_{i,j}+R\cdot \left(1-\frac{t}{T}\right)\cdot \left(2\cdot rand-1\right)\cdot x_{i,j}$$

where the updated status of $i^{th}$ pelican at $j^{th}$ parameter is $X_{i,j}^{P_{2}}$, which relies on phase 2. R is a constant fixed at 0.2, and the radius of close vicinity around $x_{i,j}$ is R⋅(1−t/T). Now, t and T are the iteration and the maximum iteration counter. In this phase, the concept of effective updating, as shown in Equation (8), is used to decide whether the new location of the pelican needs to be rejected or accepted.

(8) $$X_{i}=\left\{\begin{array}{ll} X_{i}^{P_{2}}, & if\;F_{i}^{P_{2}}<F_{i}\\ X_{i}, & else \end{array}\right.$$

where the value of the objective function attained during phase 2 is $F_{i}^{P_{2}}$, while the new position of the $i^{th}$ pelican after phase 2 is $X_{i}^{P_{2}}$.

The first location of the population in the search range is allocated consistently to increase the search effectiveness and global search capability of the algorithm. Different from the typical POA technique that randomly initializes the population and may decline the population diversity, it uses logistic-sine chaotic mapping for the initialization population.

The logistic-sine chaotic map fuses the features of sine and logistic mapping. This can differ from logical and sinusoidal maps since it has large chaotic intervals. It ensures the randomness of individuals within the population by generating a primary population, which can be allocated at random through the logistic sine map. Equation (11) through (13) provides the arithmetical formulation for the logistic-sine mapping, the logistic mapping, and the sine mapping, correspondingly.

(9) $$Z_{i+1}=\mu Z_{i}\left(1-Z_{i}\right)$$

(10) $$Z_{i+1}=sin\left(\pi Z_{j}\right)$$

(11) $$Z_{i+1}=\left(\mu Z_{i}\left(1-Z_{i}\right)+\frac{\left(4-\mu \right)\sin\left(\pi Z_{i}\right)}{4}\right)\left(mod1\right)$$

Here, a sequence of numbers produced at random is Z and a multiplier of chaos is μ. Sine and logistic mappings are defined explicitly in Equations (11) and (12), correspondingly. The mathematical representation of logistic-sine mapping is shown in Equation (13).

(12) $$x_{i}=l_{b}+\left(u_{b}-l_{b}\right)*Z_{j}$$

According to the Levy flight-based jumps, the balance between exploitation and exploration is obtained, and it enables individuals to catch a number of fish during hunting. The non-Gaussian stochastic model is Levy’s flight, otherwise called Levy motion, which carries out random walking attained in Levy stabilization. This distribution describes the power law equation $L\left(s\right)∼|s{|}^{-l-\beta }$, in which 0<β<2 signifies an index and s means the step length. The step length can be determined by

(13) $$s=\frac{u}{|v{|}^{\frac{1}{\beta }}}$$

where uniform distribution serves as a source for u and v.

(14) $$uN\left(0,\sigma_{u}^{2}\right),vN\left(0,\sigma_{v}^{2}\right)$$

where

(15) $$\sigma_{u}={\left\{\frac{\Gamma \left(1+\beta \right)\times \sin\left(\frac{\pi \beta }{2}\right)}{\Gamma \left(\frac{1+\beta }{2}\right)\times \beta \times 2\left(\frac{\beta -1}{2}\right)}\right\}}^{\frac{1}{\beta }},\sigma_{v}=1$$

while the Levy function is added in this work. The updated location of the pelican can be computed using the following expression:

(16) $$X_{j}=\left\{\begin{array}{l} X_{i}^{P_{2}}+\alpha ⊕Levy,\;if\;F_{i}^{P_{2}}<F_{i}\\ X_{i},\;else \end{array}\right.$$

where

(17) $$\alpha =0.01\times s\times \left(X_{i}^{P_{2}}-X_{besi}\right)$$

The EPOA method refers to an FF to obtain higher proficiency in classification. It determines the positive integer to signify the best efficiency of the solution candidate. The failure of the classifier error rate can be assumed as an FF.

(18) $$fitness\left(x_{i}\right)=ClassifierErrorRate\left(x_{i}\right)\;=\frac{no.of\;misclassified\;samples}{Total\;no.of\;samples}*100$$

### 3.3. Stage III: ANFIS Classifier

**Figure 2:**
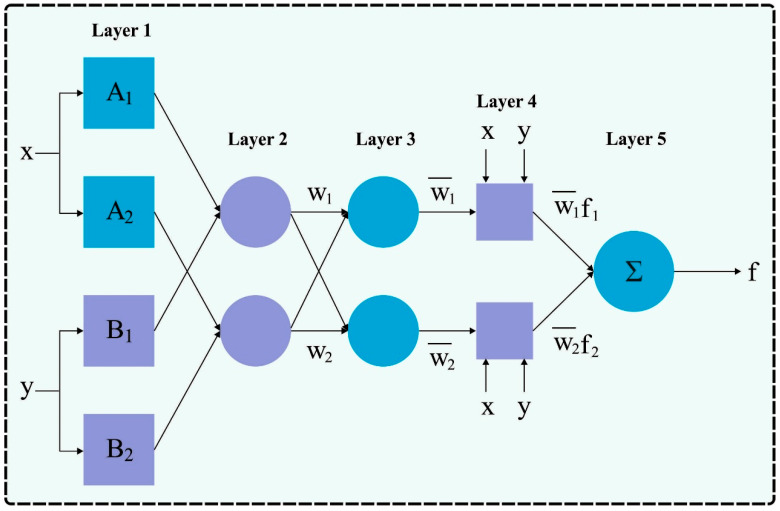
ANFIS structure.

For the classification process, the ANFIS model can be used. The neuro-fuzzy model is ANFIS, which exploits the implication features of FL and the learning capability of ANN. This can have considerable benefits as it uses the strengths of both ANNs and FL [24]. The antecedent and the conclusion are two parts of ANFIS. IF-THEN fuzzy procedures determine whether both parts could be mutually interconnected. Figure 2 demonstrates the infrastructure of ANFIS. The ANFIS comprises five layers.

Layer 1 is named the fuzzification layer. Here, the MF is utilized, and a fuzzy set is attained from the input value using MF. The parameters in the MF architecture have been named antecedent parameters. The shapes of MF were designed in accordance with the values of these parameters. The membership function has a membership degree. The membership degree takes a value within [0, 1]. When the generalization Bell function can be utilized as an MF, the membership degree is evaluated via the following expression:

(19) $$\mu_{A_{i}}\left(x\right)=gbellmf\left(x,a,b,c\right)=\frac{1}{1+{\left|\frac{x-c}{a}\right|}^{2b}}$$

(20) $$O_{1}^{1}=\mu_{A_{i}}\left(x\right)$$

Layer 2 is named as a rule layer. Firing strength is found for all the rules. In the prior layer, the membership values found are utilized in the computation of firing strength. By multiplying the membership value, the firing strength (w) value is found using Equation (21):

(21) $$O_{1}^{2}=w_{i}=\mu_{A_{i}}\left(x\right)*\mu_{B_{i}}\left(y\right),i=1,2.$$

Layer 3 can be called the normalization layer. Normalized firing strength is evaluated using all the rules. Normalized firing strength $\bar{w_{i}}$ is evaluated via the following equation:

(22) $$O_{1}^{3}=\bar{w_{i}}=\frac{w_{i}}{w_{1}+w_{2}+w_{3}+w_{4}},i\in \left\{1,2,3,4\right\}$$

Layer 4 is referred to as the defuzzification layer. The resultant values of all the rules are evaluated. A first-order polynomial and normalized firing strength are utilized for calculating the output for all the rules.

(23) $$O_{1}^{4}=\bar{w_{i}}f_{i}=\bar{w_{i}}\left(p_{i}x+q_{i}y+r_{i}\right)$$

Layer 5 is named the summation layer. In the defuzzification layer, the original outcome of ANFIS can be accomplished by summing the output for all the rules.

(24) $$O_{1}^{5}=\sum_{i}\bar{w_{i}}f_{i}=\frac{\Sigma_{i}w_{i}f_{i}}{\Sigma_{i}w_{i}}$$

## 4. Results and Discussion

The proposed model is simulated using the Python 3.8.5 tool. The proposed model is experimented on PC i5-8600k, GeForce 1050Ti 4 GB, 16 GB RAM, 250 GB SSD, and 1 TB HDD. In this section, the investigational analysis of the EPOADL-MNC technique could be applied to the histopathological image dataset [25], including 150 images, as represented in Table 1. The database includes 75 mitosis and 75 non-mitosis images.

**Figure 3:**
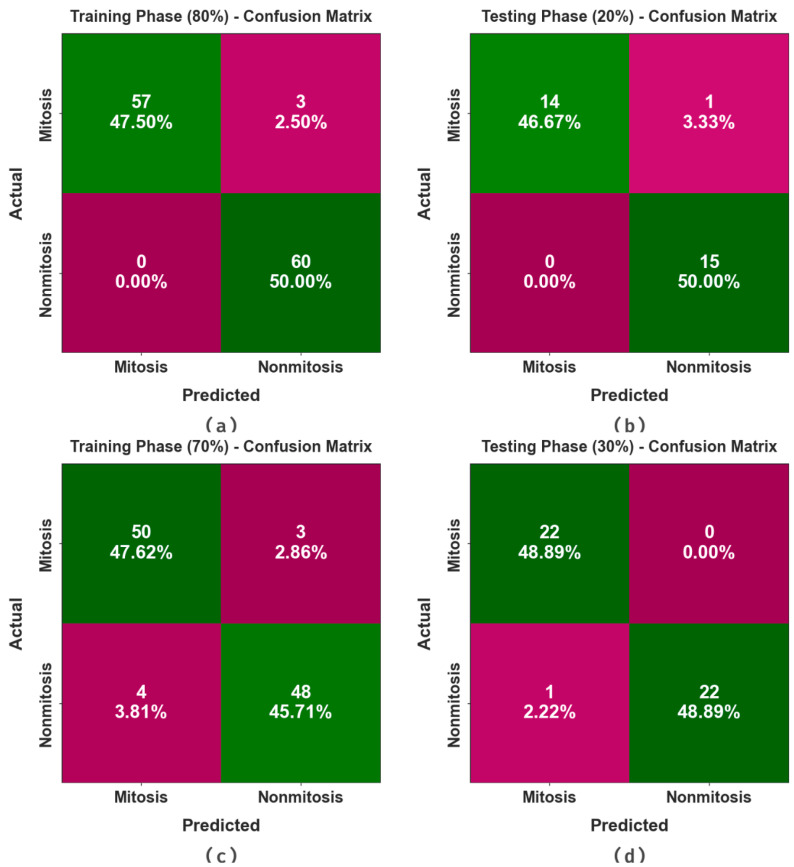
(**a**,**b**) Confusion matrices with 80:20 of TR phase/TS phase and (**c**,**d**) 70:30 of TR phase/TS phase.

Figure 3 illustrates the confusion matrices made by the EPOADL-MNC algorithm with 80:20 and 70:30 of the TR phase/TS phase. The outcomes demonstrate the efficient detection of the mitosis and non-mitosis samples in all classes.

**Figure 4:**
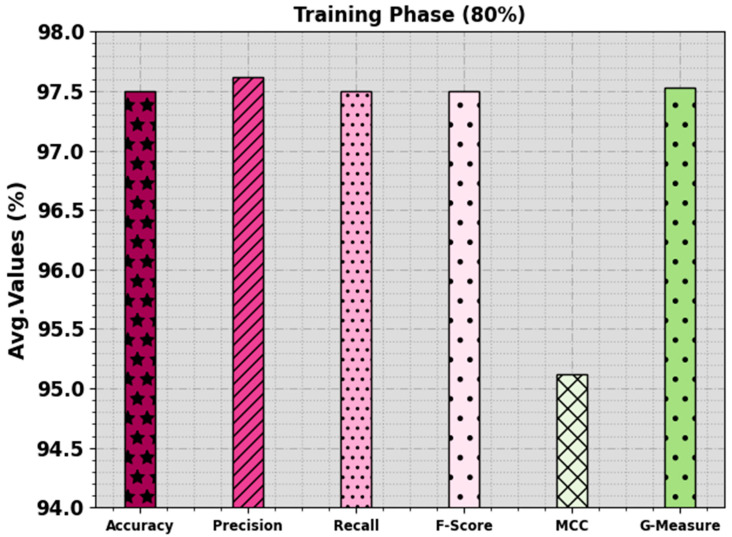
Average of EPOADL-MNC model with 80% of TR phase.

**Figure 5:**
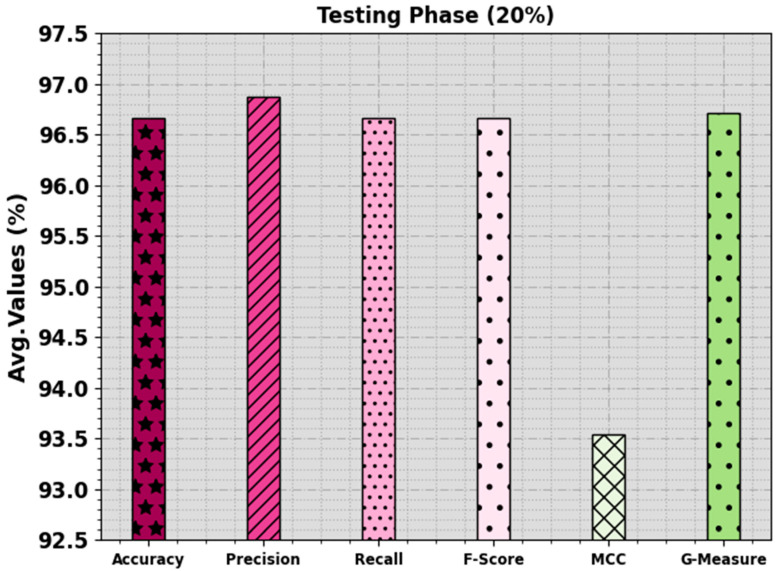
Average of EPOADL-MNC model with 20% of TS phase.

In Table 2 and Figure 4 and Figure 5, the mitosis nuclei classifier outcomes of the EPOADL-MNC technique with 80:20 of the TR phase/TS phase are shown. The simulation outcome signifies that the EPOADL-MNC system properly recognizes the mitosis and non-mitosis samples. On 80% of the TR phase, the EPOADL-MNC technique classifies mitosis samples with an $accu_{y}$ of 95.00%, $prec_{n}$ of 100.00%, $reca_{l}$ of 95.00%, $F_{score}$ of 97.44%, MCC of 95.12%, and $G_{measure}$ of 97.47%. Moreover, on 20% of the TS phase, the EPOADL-MNC approach classifies mitosis samples with an $accu_{y}$ of 93.33%, $prec_{n}$ of 100.00%, $reca_{l}$ of 93.33%, $F_{score}$ of 96.55%, MCC of 93.54%, and $G_{measure}$ of 96.61%.

**Figure 6:**
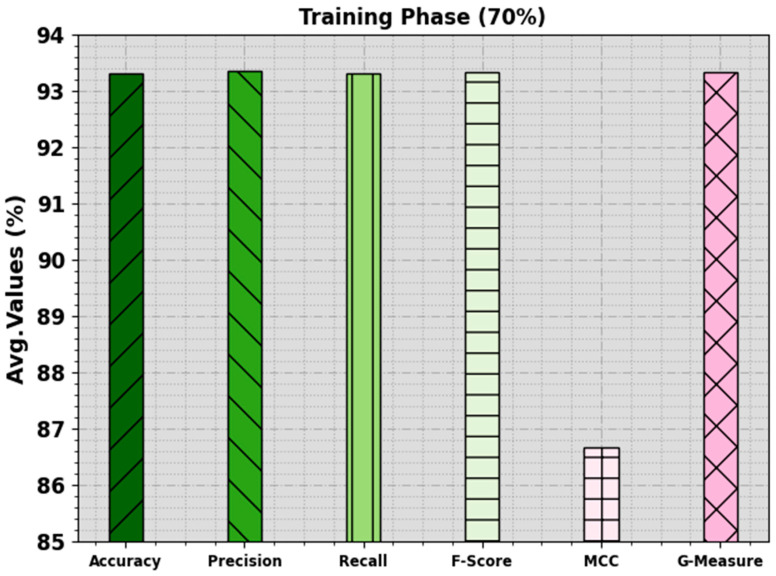
Average of EPOADL-MNC model in 70% of TR phase.

**Figure 7:**
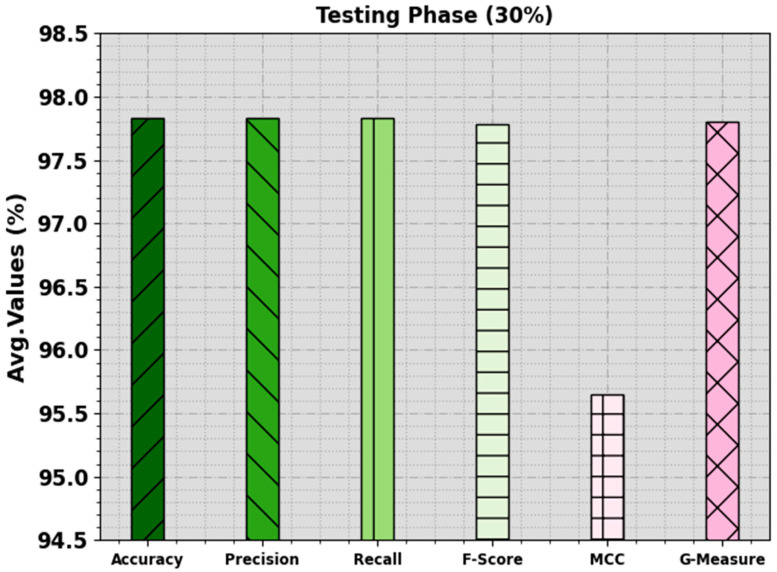
Average of EPOADL-MNC algorithm at 30% of TS phase.

In Table 3 and Figure 6 and Figure 7, the mitosis nuclei classifier analysis of the EPOADL-MNC methodology with 70:30 of the TR phase/TS phase is given. The achieved values indicate that the EPOADL-MNC system correctly recognizes the mitosis and non-mitosis samples. On 70% of the TR phase, the EPOADL-MNC model classifies mitosis samples with an $accu_{y}$ of 94.34%, $prec_{n}$ of 92.59%, $reca_{l}$ of 94.34%, $F_{score}$ of 93.46%, MCC of 86.68%, and $G_{measure}$ of 93.46%. According to 30% of the TS phase, the EPOADL-MNC method classifies mitosis samples with an $accu_{y}$ of 100%, $prec_{n}$ of 95.65%, $reca_{l}$ of 100%, $F_{score}$ of 97.78%, MCC of 95.65%, and $G_{measure}$ of 97.80%.

**Figure 8:**
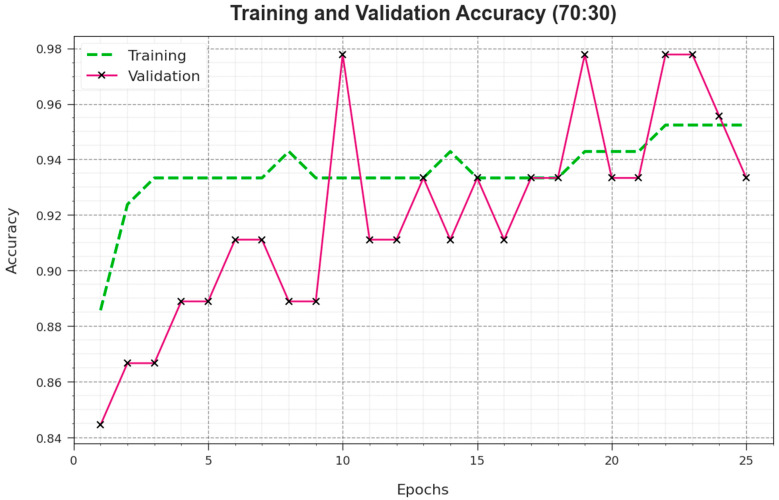
$Accu_{y}$ curve of EPOADL-MNC model with 70:30 of TR phase/TS phase.

To compute the effectiveness of the EPOADL-MNC system in 70:30 of the TR phase/TS phase, $accu_{y}$ curves of TRA and TES are described and depicted in Figure 8. These $accu_{y}$ curves, TR and TS, depict the analysis of the EPOADL-MNC method over numerous epochs. This figure provides important details about the learning processes and generalization capabilities of the EPOADL-MNC technique. A higher epoch count means that the $accu_{y}$ curves of TES and TRA are enhanced. The EPOADL-MNC method provides a higher testing accuracy that can identify the patterns in the datasets of TRA and TES.

**Figure 9:**
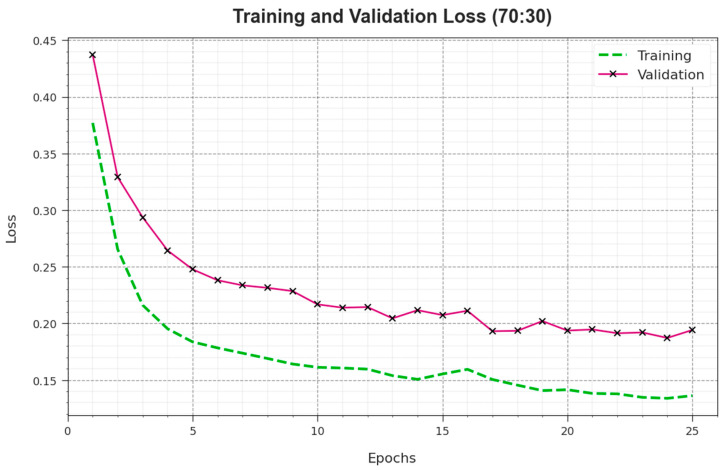
Loss curve of EPOADL-MNC algorithm in 70:30 of TR phase/TS phase.

Figure 9 displays the comprehensive TRA and TES loss values of the EPOADL-MNC system at 70:30 of the TR phase/TS phase over epochs. This TRA loss displays that the model loss can be lesser over epochs. Generally, the loss values can be decreased as the model varies the weight to decrease the prediction error for the data of TRA and TES. These loss curves pointed out the level of how a model is fitted to the training database. This could be seen from the TRA and TES loss, which was progressively minimized and demonstrated that the EPOADL-MNC approach efficaciously learned the patterns shown in the TRA and TES data, as well as adjusted the parameters for lessening the differences between the original and predictive training labels.

**Figure 10:**
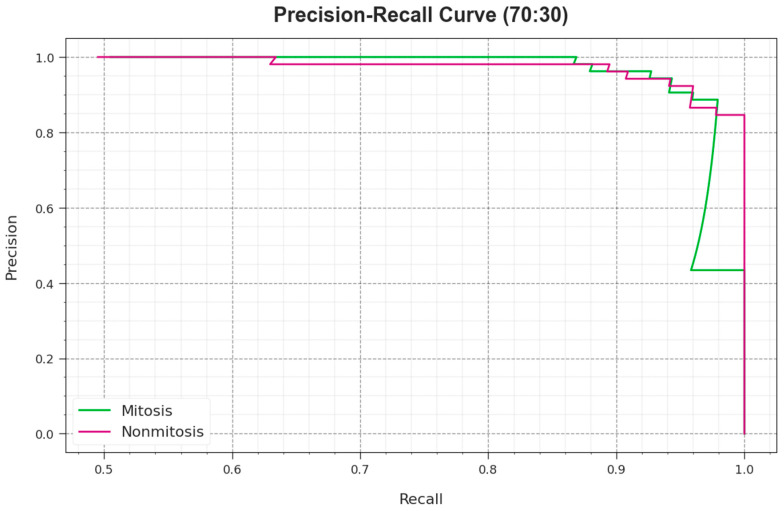
PR curve of EPOADL-MNC system at 70:30 of TR phase/TS phase.

The PR effectiveness of the EPOADL-MNC approach in 70:30 of the TR phase/TS phase can be represented via plotting PR, as represented in Figure 10. These obtained values pointed to the EPOADL-MNC system obtaining higher PR analysis in each class. This figure shows that the model learns to distinguish numerous class labels. The EPOADL-MNC algorithm accomplishes improved outcomes with the identification of positive instances and reduced false positives.

**Figure 11:**
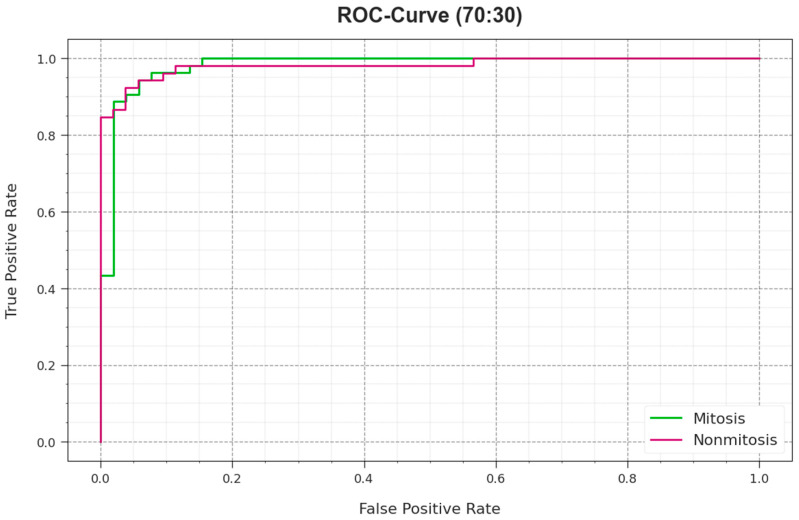
ROC curve of EPOADL-MNC model at 70:30 of TR phase/TS phase.

Figure 11 displays the ROC curves given by the EPOADL-MNC algorithm in 70:30 of the TR phase/TS phase, which can provide the capability to differentiate each class. This figure indicates valued insights into trade-offs amongst the FPR and TPR rates over numerous classification epochs and thresholds. This provides the correct predicted outcome of EPOADL-MNC methodology in the categorization of distinct classes.

**Figure 12:**
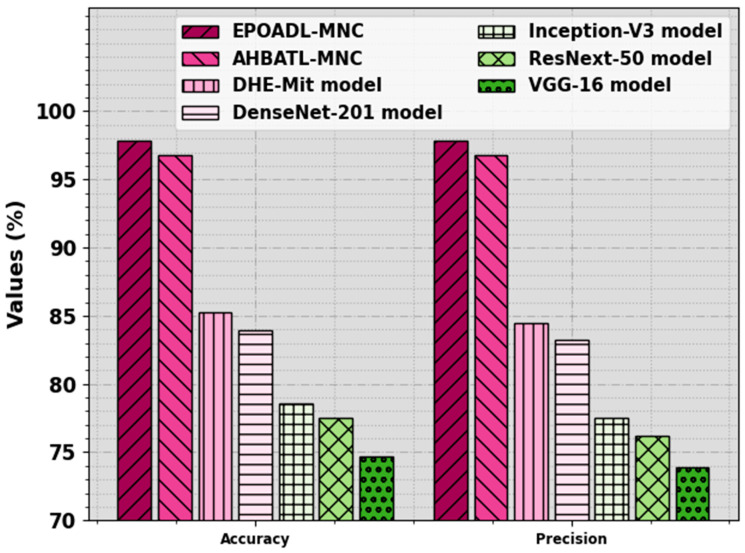
$Accu_{y}$ and $prec_{n}$ outcome of EPOADL-MNC algorithm with recent approaches.

The enhanced effectiveness of the EPOADL-MNC methodology on the mitotic nuclei cell classification process is compared with other models in Table 4 [26]. Figure 12 represents the comparison analysis of the EPOADL-MNC system with other methodologies for $accu_{y}$ and $prec_{n}$. The attained outcome stated that the EPOADL-MNC model gains better performance over other systems. Based on $accu_{y}$, the EPOADL-MNC technique offers an increased $accu_{y}$ of 97.83% while the AHBATL-MNC, DHE-Mit, DenseNet201, Inceptionv3, ResNext50, and VGG16 methodologies provide decreased $accu_{y}$ values of 96.77%, 85.23%, 83.96%, 78.54%, 77.48%, and 74.72%, respectively. Also, based on $prec_{n}$, the EPOADL-MNC approach attains a higher $prec_{n}$ of 97.83% while the AHBATL-MNC, DHE-Mit, DenseNet201, Inceptionv3, ResNext50, and VGG16 systems provide decreased $prec_{n}$ values of 96.77%, 84.45%, 83.20%, 77.51%, 76.20%, and 73.93%, respectively.

**Figure 13:**
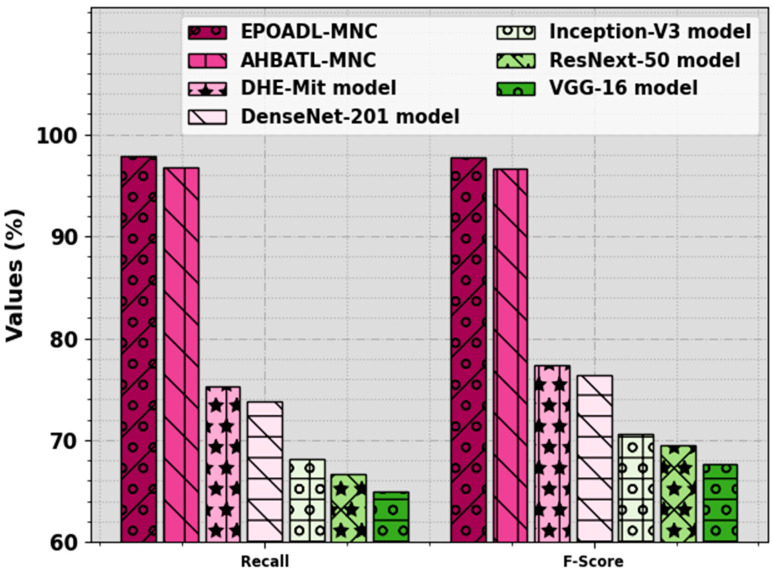
$Reca_{l}$ and $F_{score}$ outcomes of EPOADL-MNC algorithm with recent approaches.

Figure 13 signifies the comparative outcome of the EPOADL-MNC approach with recent systems in terms of $reca_{l}$ and $F_{score}$. The outcomes implied that the EPOADL-MNC technique attains improved performance over other approaches. According to $reca_{l}$, the EPOADL-MNC approach offers a higher $reca_{l}$ of 97.83% while the AHBATL-MNC, DHE-Mit, DenseNet201, Inceptionv3, ResNext50, and VGG16 systems provide lesser $reca_{l}$ values of 96.77%, 75.26%, 73.85%, 68.18%, 66.73%, and 65%, correspondingly. Moreover, based on $F_{score}$, the EPOADL-MNC approach offers an enhanced $F_{score}$ of 97.78%, while the AHBATL-MNC, DHE-Mit, DenseNet201, Inceptionv3, ResNext50, and VGG16 algorithms provide minimal $F_{score}$ values of 96.77%, 77.33%, 76.38%, 70.64%, 69.49%, and 67.66%, correspondingly.

These performances confirmed the optimum outcome of the EPOADL-MNC system in the classification of mitotic cells. The maximum solution of the EPOADL-MNC method can be attributed to its holistic model which optimizes several facets of the mitotic nuclei classification model. By seamlessly combining DL, advanced feature extraction with ShuffleNet, hyperparameter tuning by EPOA, and precise classification utilizing the ANFIS approach, the EPOADL-MNC systematically addresses the challenges of breast histopathology image analysis. The fine-tuned hyperparameters, specially tailored to the ShuffleNet approach, ensure that relevant features can be effectually extracted. This is integrated with the capability of ANFIS to offer accurate classifications and performances in a synergistic group of modules that collectively contribute to a remarkable 97.83% accuracy rate, surpassing recent DL approaches and underlining the model’s comprehensive efficiency in mitotic nuclei detection and classification.

## 5. Conclusions

In this article, we have designed the EPOADL-MNC technique for automated mitotic nuclei classification. The aim of the EPOADL-MNC system is presented in the examination of the histopathology images for the classification of MCs and non-MCs. In the presented EPOADL-MNC technique, the ShuffleNet feature extractor, EPOA-based parameter tuning, and ANFIS classifier are involved. Primarily, the ShuffleNet model can be employed for processing feature extraction. For the hyperparameter tuning procedure, the EPOADL-MNC technique makes use of the EPOA approach to vary the hyperparameters of the ShuffleNet model. Finally, the ANFIS approach has been employed for the detection and classification of mitotic cell nuclei on HI. The series of simulations took place to validate the superior detection outcomes of the EPOADL-MNC model. The comprehensive outcomes emphasized the better outcome of the EPOADL-MNC system related to DL methods. In future work, the EPOADL-MNC model can be extended to accommodate multi-class classification to address a wider range of cellular structures in histopathology images. Additionally, exploring the integration of real-time image acquisition systems for more immediate and dynamic breast cancer diagnosis is a promising avenue, enhancing the model’s applicability in clinical settings.

## Figures and Tables

**Table 1:** Details on database.

Class	No. of Images
Mitosis	75
Non-mitosis	75
Total No. of Images	150

**Table 2:** Mitosis nuclei classifier analysis of EPOADL-MNC system in 80:20 of TR phase/TS phase.

Class	$Accu_{y}$	$Prec_{n}$	$Reca_{l}$	$F_{Score}$	MCC	$G_{Measure}$
TR Phase (80%)						
Mitosis	95.00	100.00	95.00	97.44	95.12	97.47
Non-mitosis	100.00	95.24	100.00	97.56	95.12	97.59
Average	97.50	97.62	97.50	97.50	95.12	97.53
TS Phase (20%)						
Mitosis	93.33	100.00	93.33	96.55	93.54	96.61
Non-mitosis	100.00	93.75	100.00	96.77	93.54	96.82
Average	96.67	96.88	96.67	96.66	93.54	96.72

**Table 3:** Mitosis nuclei classifier outcome of EPOADL-MNC model at 80:20 of TR phase/TS phase.

Class	$Accu_{y}$	$Prec_{n}$	$Reca_{l}$	$F_{Score}$	MCC	$G_{Measure}$
TR Phase (70%)						
Mitosis	94.34	92.59	94.34	93.46	86.68	93.46
Non-mitosis	92.31	94.12	92.31	93.20	86.68	93.21
Average	93.32	93.36	93.32	93.33	86.68	93.34
TS Phase (30%)						
Mitosis	100.00	95.65	100.00	97.78	95.65	97.80
Non-mitosis	95.65	100.00	95.65	97.78	95.65	97.80
Average	97.83	97.83	97.83	97.78	95.65	97.80

**Table 4:** Comparison outcome of EPOADL-MNC algorithm with recent models.

Methods	$Accu_{y}$	$Prec_{n}$	$Reca_{l}$	$F_{Score}$
EPOADL-MNC	97.83	97.83	97.83	97.78
AHBATL-MNC	96.77	96.77	96.77	96.67
DHE-Mit	85.23	84.45	75.26	77.33
DenseNet201	83.96	83.20	73.85	76.38
Inception-V3	78.54	77.51	68.18	70.64
ResNext50	77.48	76.20	66.73	69.49
VGG-16	74.72	73.93	65.00	67.66

## Data Availability

Data sharing does not apply to this article as no datasets were produced in the current study.
